# Maternal Dietary Yeast Hydrolysate Alters Milk Composition and Influences Metabolic Status and Gut Microbiota in Dezhou Donkey Foals

**DOI:** 10.3390/ijms27167171

**Published:** 2026-08-11

**Authors:** Zhangxinhan Wen, Zuowei Li, Tianzheng Wang, Yaru Wang, Pengshuai Li, Yuhan Yin, Boying Dong, Yulong Feng, Honglei Qu, Qiugang Ma, Haihua Zhang, Shimeng Huang

**Affiliations:** 1 State Key Laboratory of Animal Nutrition and Feeding, College of Animal Science and Technology, China Agricultural University, Beijing 100193, China; 2 Laboratory of Feed Grain Safety and Healthy Poultry Farming, Beijing Jingwa Agricultural Science and Technology Innovation Center, Beijing 101206, China; 3 Hebei Key Laboratory of Specialty Animal Germplasm Resources Exploration and Innovation, College of Animal Science and Technology, Hebei Normal University of Science and Technology, Qinhuangdao 066004, China; 4 National Engineering Research Center for Gelatin-Based Traditional Chinese Medicine, Dong-E-E-Jiao Co., Ltd., Liaocheng 252201, China

**Keywords:** yeast hydrolysate, Dezhou donkey foals, milk nutrient profiles, gut microbiota, growth

## Abstract

Yeast hydrolysate (YH) has attracted increasing interest as a functional feed additive because of its potential benefits for maternal nutrition and offspring development. However, its effects on milk composition and the physiological responses of donkey foals remain poorly understood. This study evaluated the effects of dietary YH supplementation in lactating Dezhou jennies on milk composition, as well as growth performance, serum biochemical parameters, and fecal microbiota in their foals. Sixteen healthy jennies and their foals were randomly assigned to a control group (OCON) or a YH-supplemented group (OYE; 0.5 g/kg diet) for 60 days. Maternal dietary YH supplementation significantly increased milk fat and milk protein contents. Foals in the OYE group showed a tendency toward greater back skin thickness, although the difference was not statistically significant, while body weight and neck skin thickness were unaffected. Serum biochemical analysis showed increased HDL-C and total cholesterol concentrations together with decreased triglyceride and LDL-C concentrations in the OYE group. In addition, serum ALT activity was significantly decreased, whereas AST levels were significantly increased. Fecal microbiota analysis indicated that YH supplementation did not significantly affect overall microbial diversity but altered the relative abundance of several bacterial taxa, including Prevotella, Alistipes, and Olsenella. Overall, maternal dietary YH supplementation was associated with changes in milk composition, selected serum biochemical parameters, and the relative abundance of specific fecal bacterial taxa in donkey foals. However, no significant improvement in offspring growth performance was observed, and the underlying mechanisms require further investigation.

## 1. Introduction

The donkey industry is playing an increasingly important role in modern animal agriculture due to its contributions to traditional medicine, specialty dairy products, and rural economic development [1,2,3]. The establishment of healthy and sustainable donkey production systems is therefore of considerable importance [2]. Among the factors influencing production efficiency, early-life growth and health of donkey foals are critical determinants of lifetime productivity, disease resistance, and overall breeding performance [4,5,6]. Nutritional interventions during the maternal and neonatal periods have been recognized as particularly effective strategies for promoting foal development, improving animal welfare, and enhancing long-term production outcomes [6,7,8].

Yeast hydrolysate (YH), a functional feed additive produced through the enzymatic hydrolysis of Saccharomyces cerevisiae, is rich in a wide range of bioactive components, including mannan oligosaccharides, β-glucans, nucleotides, peptides, amino acids, and B-complex vitamins [9,10,11]. These functional constituents confer multifaceted biological activities, such as modulation of intestinal microbiota composition, enhancement of nutrient digestion and absorption, reinforcement of intestinal barrier integrity, regulation of immune and inflammatory responses, and improvement in antioxidant capacity [9,10,11,12,13,14,15]. Owing to these properties, YH has attracted increasing attention as a safe and effective nutritional strategy in livestock production, particularly in the context of global efforts to reduce or replace antibiotic growth promoters [3,10,11]. Extensive studies have demonstrated that dietary supplementation with YH or its functional fractions improves intestinal morphology, reduces diarrhea incidence, attenuates pro-inflammatory cytokines production, optimizes lipid and protein metabolism, and enhances growth performance, milk yield, and milk quality [3,9,10,11,12,13,14,15]. These findings collectively highlight the integrative role of YH in coordinating the “gut–immune–metabolism” axis. Importantly, YH and its derivatives are generally recognized as safe and non-toxic, further supporting their application as functional feed additives in modern, sustainable livestock production systems. Despite these advances, research on the application of YH in equines—particularly in donkeys—remains limited. This knowledge gap is notable given the increasing importance of Dezhou donkeys in the food and pharmaceutical industries, as well as the critical role of early-life nutrition in determining foal health, metabolic programming, and long-term productivity [6,7,8]. Moreover, maternal nutrition during lactation directly influences milk composition, which serves as the primary source of nutrients and bioactive factors for nursing foals and plays a pivotal role in shaping early growth, metabolic status, and gut microbial colonization.

We therefore hypothesized that maternal dietary supplementation with yeast hydrolysate would improve milk nutritional quality and, in turn, promote growth performance, metabolic homeostasis, and the intestinal health of nursing donkey foals through coordinated regulation of maternal metabolism and foal gut microbiota. Accordingly, the objective of this study was to systematically evaluate the effects of yeast hydrolysate supplementation in lactating Dezhou donkeys on milk composition, foal growth performance, blood biochemical indices, and fecal microbial communities. The findings of this study aim to provide a scientific basis for precision nutritional strategies to enhance foal health, improve animal welfare, and support the sustainable development of the donkey industry.

## 2. Results

### 2.1. Growth Performance of Dezhou Donkey Foals

**Figure 1:**
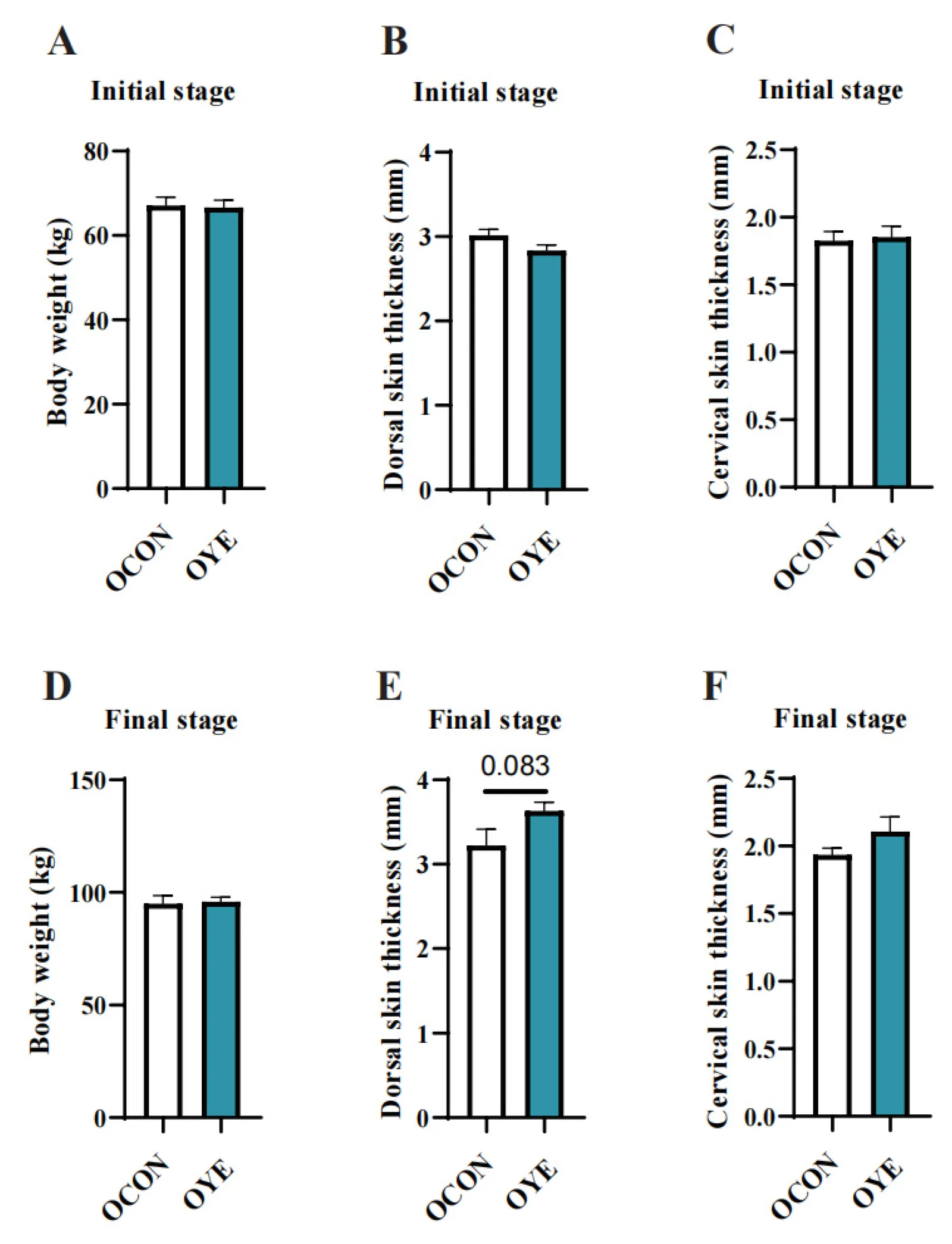
Effects of Maternal Dietary Yeast Hydrolysate Supplementation on Growth Performance and Skin Development of Dezhou Donkey Foals. (**A**) Initial body weight; (**B**) Initial dorsal skin thickness; (**C**) Initial cervical skin thickness; (**D**) Final body weight; (**E**) Final dorsal skin thickness; (**F**) Final cervical skin thickness. Data (**A**–**F**) were analyzed using analysis of variance (ANOVA). Data are presented as mean ± standard deviation (*n* = 8 per group).

Data on the growth performance of donkey foals are presented in Figure 1. At the end of the experimental period, foals in the OYE group showed a tendency toward increased back skin thickness compared with those in the OCON group (*p* = 0.08; Figure 1E). However, no significant differences were observed between the two groups in final body weight (*p* > 0.05; Figure 1D) or neck skin thickness (*p* > 0.05; Figure 1F).

### 2.2. Blood Biochemical Indices of Donkey Foals

**Figure 2:**
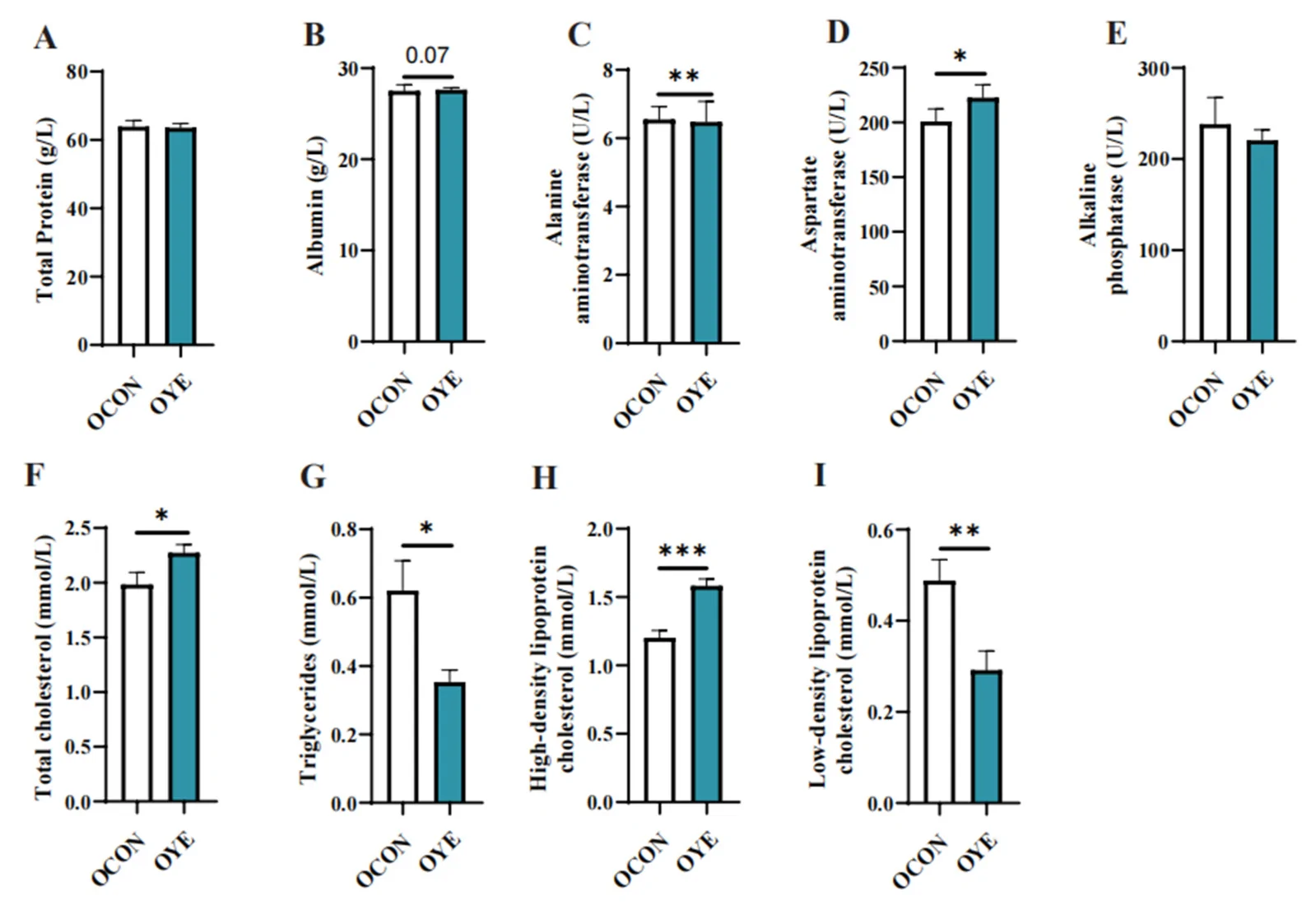
Effects of Maternal Dietary Yeast Hydrolysate Supplementation on blood biochemical indices of Dezhou Donkey Foals. (**A**) Total Protein (g/L); (**B**) Albumin (g/L); (**C**) Alanine aminotransferase (U/L); (**D**) Aspartate aminotransferase (U/L); (**E**) Alkaline phosphatase (U/L); (**F**) Total cholesterol (mmol/L); (**G**) Triglycerides (mmol/L); (**H**) High-density lipoprotein cholesterol (mmol/L); (**I**) Low-density lipoprotein cholesterol (mmol/L). Data are presented as mean ± standard deviation (*n* = 8 per group). * *p* < 0.05, ** *p* < 0.01, *** *p* < 0.001.

The blood biochemical parameters of donkey foals are presented in Figure 2. Serum ALB concentration tended to be higher in the OYE group than in the OCON group; however, the difference did not reach statistical significance (*p* = 0.07; Figure 2B). With respect to liver function-related biochemical markers, ALT activity was significantly lower in the OYE group than in the OCON group (*p* < 0.01; Figure 2C), whereas serum AST levels were significantly higher (*p* < 0.05; Figure 2D). ALP activity also tended to be lower in the OYE group, although this difference was not statistically significant (Figure 2E). Regarding lipid metabolism, TC concentration was significantly increased in the OYE group compared with the OCON group (*p* < 0.05; Figure 2F), whereas TG levels were significantly reduced (*p* < 0.05; Figure 2G). In addition, HDL-C concentration was markedly elevated in the OYE group (*p* < 0.001; Figure 2H), while LDL-C concentration was significantly decreased relative to the OCON group (*p* < 0.01; Figure 2I).

### 2.3. Milk Composition and Milk Metabolites of Maternal Donkeys

**Figure 3:**
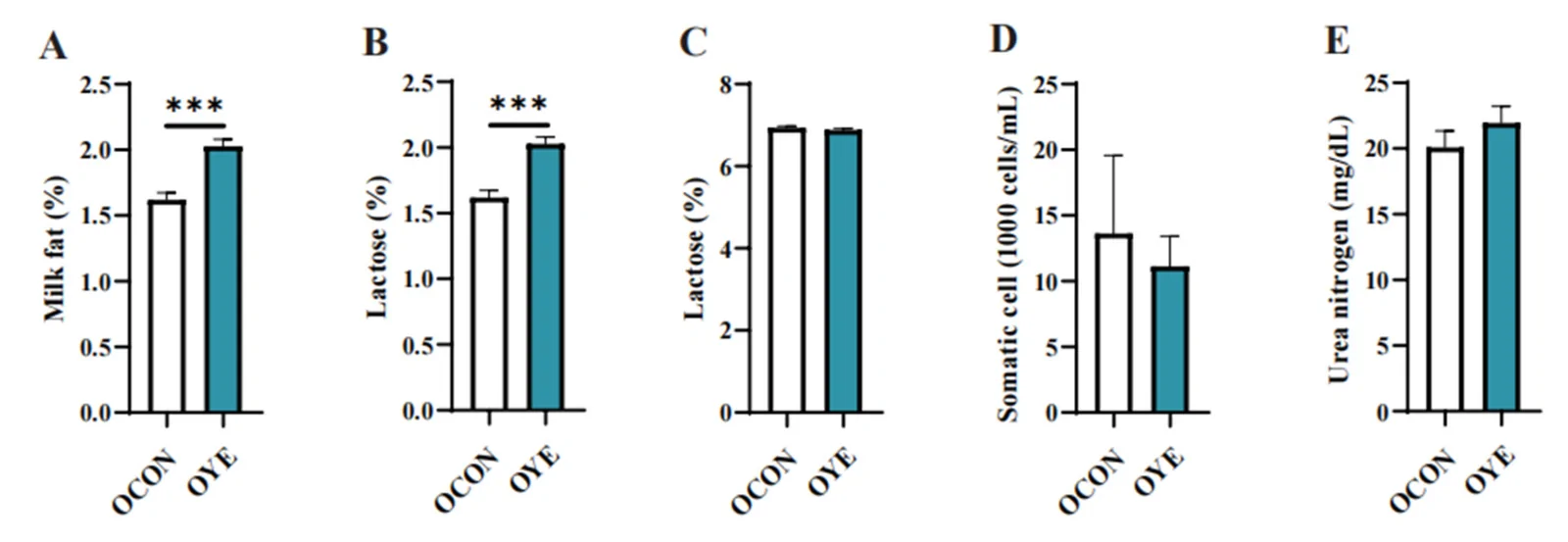
Effects of Maternal Dietary Yeast Hydrolysate Supplementation on Milk Nutritional Composition in Lactating Dezhou Donkeys. (**A**) Milk fat; (**B**) Milk protein; (**C**) Lactose; (**D**) Somatic cell count; (**E**) Urea nitrogen. Differences in data (**A**–**E**) were tested by analysis of variance (ANOVA). Data are presented as mean ± standard deviation (*n* = 8 per group). *** *p* < 0.001.

The effects of yeast hydrolysate supplementation on the milk composition of maternal donkeys are presented in Figure 3. Compared to the OCON group, milk fat and milk protein contents in the OYE group were markedly increased (*p* < 0.001; Figure 3A,B). The somatic cell count in milk from the OYE group was numerically lower than that in the OCON group; however, this difference did not reach statistical significance (*p* > 0.05; Figure 3D). No significant differences were observed between the two groups in milk lactose or urea nitrogen concentrations (*p* > 0.05; Figure 3C,E).

### 2.4. Fecal Microbial Community of Maternal Donkeys

**Figure 4:**
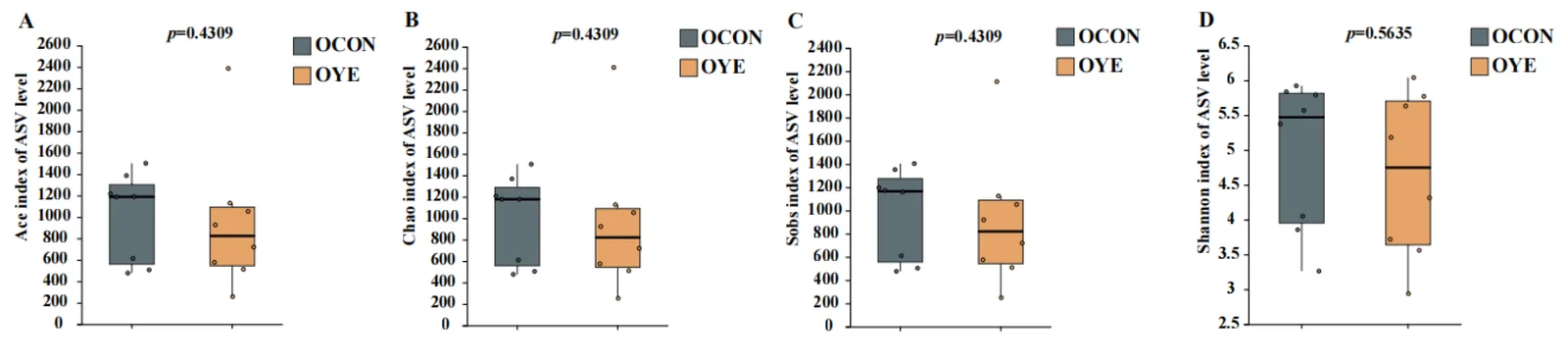
Effects of Maternal Dietary Yeast Hydrolysate Supplementation on Fecal Microbial Alpha Diversity of Dezhou Donkey Foals. (**A**) Alpha diversity indices (ACE); (**B**) Alpha diversity indices (Chao); (**C**) Alpha diversity indices (Sobs); (**D**) Alpha diversity indices (Shannon). Data are presented as mean ± standard deviation (*n* = 8 per group).

**Figure 5:**
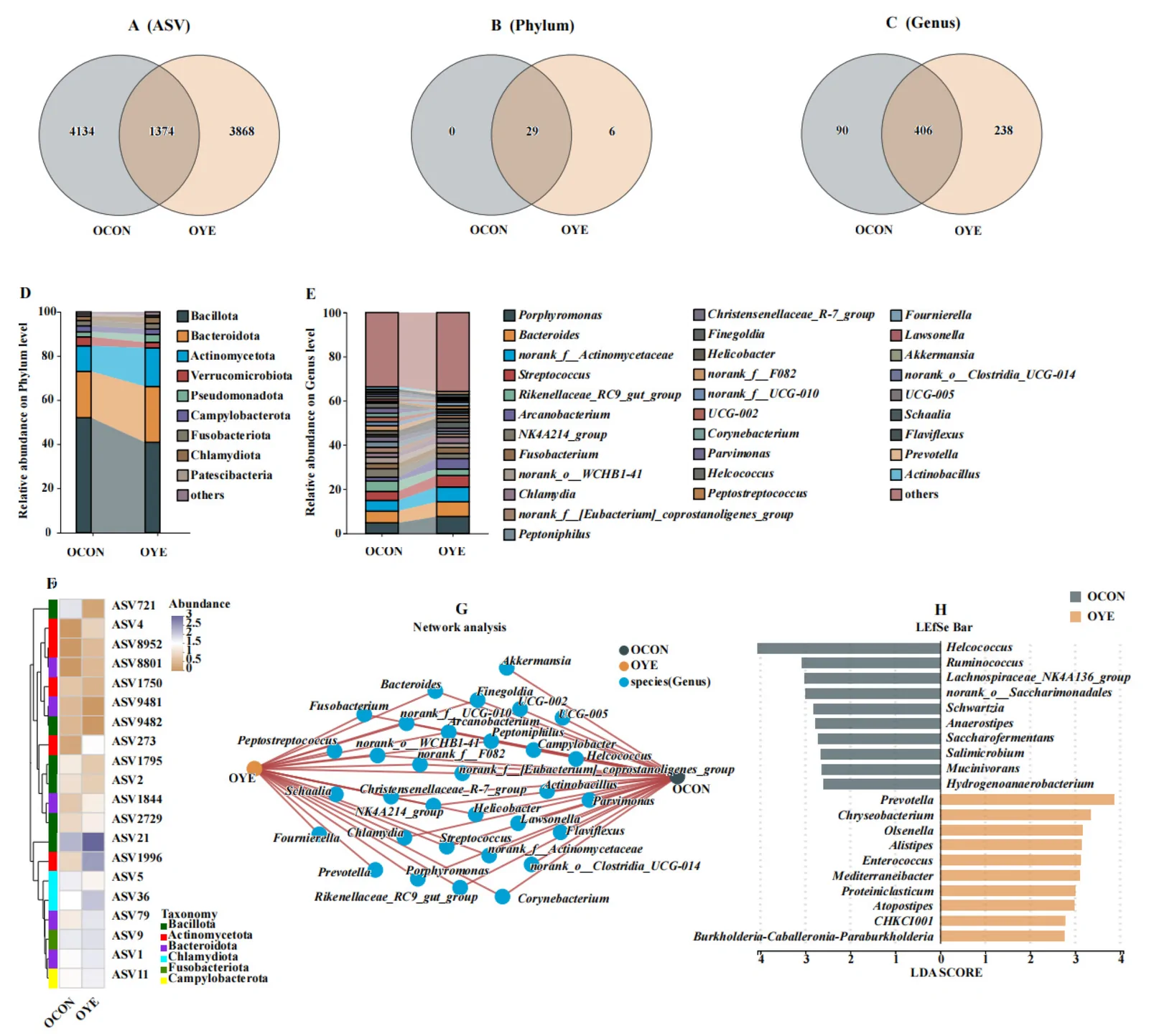
Effects of Maternal Dietary Yeast Hydrolysate Supplementation on Fecal Microbial Composition of Dezhou Donkey Foals. (**A**) Venn diagram showing shared and unique amplicon sequence variants (ASVs) between the OCON and OYE groups; (**B**) Venn diagram showing shared and unique phyla between groups; (**C**) Venn diagram showing shared and unique genera between groups; (**D**) Fecal microbial community composition at the phylum level; (**E**) Fecal microbial community composition at the genus level; (**F**) Correlation heatmap of dominant ASVs in the OCON and OYE groups; (**G**) Correlation analysis between dominant genera and the OCON and OYE groups; (**H**) LEfSe analysis identifying differentially abundant taxa between groups (LDA score > 2.0, *p* < 0.05). Data are presented as mean ± standard deviation (*n* = 8 per group).

Alpha diversity indices, including ACE, Chao1, Sobs, and Shannon, did not differ significantly between the OCON and OYE groups (*p* > 0.05; Figure 4A–C), indicating that yeast hydrolysate supplementation did not markedly affect microbial richness or diversity in donkey foals. At the ASV, phylum, and genus levels, the OCON and OYE groups shared 1374 ASVs, 29 phyla, and 406 genera, respectively (Figure 5A–C), suggesting substantial overlap in fecal microbial composition, despite observable differences between groups. Microbial community composition differed markedly at both the phylum and genus levels (Figure 5D,E). At the phylum level (Figure 5D), Bacteroidota, Bacillota, Actinobacteriota, and Verrucomicrobiota were the dominant taxa in both groups. At the genus level (Figure 5E), both groups were enriched with predominant microbes, including Porphyromonas, Bacteroides, Streptococcus, Arcanobacterium, Fusobacterium, Chlamydia, and Peptoniphilus. Analysis of dominant ASVs revealed that ASV721, ASV4, ASV8952, ASV8801, ASV1750, ASV9481, ASV21, ASV1996, and ASV5 accounted for a large proportion of the microbial community in both groups (Figure 5D). Correlation analysis (Figure 5G) demonstrated that Bacteroides, Fusobacterium, Fournierella, Prevotella, and Porphyromonas were positively associated with the OYE group, whereas Akkermansia, Flaviflexus, and Actinobacillus were more strongly correlated with the OCON group. Furthermore, LEfSe analysis (LDA > 2.0, *p* < 0.05; Figure 5H) identified Helcococcus, Ruminococcus, Lachnospiraceae_NK4A136_group, Schwartzia, Anaerostipes, Saccharofermentans, Salimicrobium, Mucinivorans, and Hydrogenoanaerobacterium as significantly enriched taxa in the OCON group. In contrast, Prevotella, Chryseobacterium, Olsenella, Alistipes, and Proteiniclasticum were significantly enriched in the OYE group. Collectively, these results indicate that maternal yeast hydrolysate supplementation selectively modulated the fecal microbial structure of donkey foals, reshaping key microbial taxa without altering overall microbial diversity.

## 3. Discussion

Sustainable and welfare-oriented donkey production has gained increasing attention with the expanding utilization of donkeys in food, pharmaceutical, and functional product industries [7]. The health and growth of donkey foals during early life are critical determinants of long-term productivity [4], reproductive efficiency, and disease resistance, as early nutritional status profoundly influences intestinal maturation, metabolic programming, and immune competence [8,16]. Yeast hydrolysate, a functional feed additive derived from Saccharomyces cerevisiae, is rich in bioactive components, including mannan oligosaccharides, β-glucans, nucleotides, peptides, and free amino acids [10,17,18,19]. These compounds have been extensively reported to exert beneficial effects on intestinal barrier function, immune regulation, and nutrient metabolism. YH supplementation has been shown to improve gut morphology and barrier integrity, modulate microbial composition, enhance protein and lipid utilization, attenuate inflammatory responses, and ultimately promote growth performance and production efficiency [18,19]. Such properties render YH a promising nutritional strategy under antibiotic-reduction and antibiotic-free production systems [19,20]. However, despite growing evidence in mature livestock, studies focusing on YH application in young animals—particularly equines—remain limited, and the potential indirect effects of maternal YH supplementation on offspring health via milk-mediated pathways are poorly understood. Therefore, the present study adopted a maternal–offspring nutritional framework to investigate the effects of dietary YH supplementation in lactating Dezhou donkeys on milk composition and to evaluate the subsequent impacts on growth performance, metabolic status, and intestinal microbiota of nursing donkey foals.

With respect to growth performance, foals born to YH-supplemented dams exhibited a tendency toward increased back skin thickness, although this difference did not reach statistical significance (*p* = 0.08). Skin thickness and epithelial integrity have been proposed as indicators associated with nutritional status, protein deposition, and tissue development [21,22,23,24]. Previous studies in other species have reported that yeast-derived products may influence dermal thickness and epithelial barrier function [23,25], potentially through improved protein utilization, enhanced micronutrient availability, and modulation of gut–skin immune–metabolic signaling pathways [26,27,28]. Nevertheless, because neither body weight nor neck skin thickness differed significantly between groups, and the increase in back skin thickness did not reach statistical significance, the present data do not provide sufficient evidence to conclude that maternal YH supplementation improved offspring growth or tissue development. Future studies involving larger cohorts and longer experimental periods are warranted to determine whether these observations represent reproducible biological responses.

The alterations observed in blood biochemical parameters suggest that maternal YH supplementation may have influenced the metabolic status of nursing foals. Serum albumin concentration tended to increase in the OYE group, indicating a potential improvement in protein nutritional status, although the difference did not reach statistical significance. Albumin plays a central role in maintaining osmotic pressure and transporting nutrients and metabolites; therefore, this trend may reflect more efficient protein utilization during early growth [26,28,29,30,31]. In addition, serum ALT activity was significantly decreased, whereas AST levels were significantly increased in foals from the OYE group. Because AST is a relatively nonspecific enzyme that is present in multiple tissues, including the liver, skeletal muscle, and heart, changes in serum AST cannot be interpreted solely as an indicator of liver function. Therefore, the concurrent decrease in ALT and increase in AST does not provide sufficient evidence to conclude either an improvement or impairment of liver status. The biological significance of these findings remains unclear and warrants further investigation using additional liver-specific biomarkers and functional assessments. Moreover, the coordinated changes in lipid-related indices—characterized by decreased triglyceride and LDL-C concentrations together with a marked increase in HDL-C—suggest an alteration in lipid metabolism and cholesterol transport [32,33,34]. These observations are broadly consistent with previous studies reporting that yeast-derived functional components can influence protein and lipid metabolism in livestock [35,36,37,38]. Collectively, the present findings suggest that maternal YH supplementation may modulate selected physiological and biochemical parameters in nursing foals. However, the mechanisms underlying these responses, particularly the observed changes in liver-associated biochemical markers, remain to be elucidated.

The pronounced enhancement of milk nutritional quality provides a key mechanistic link between maternal YH supplementation and the observed improvements in foal metabolic outcomes. In the present study, milk fat and milk protein contents were significantly increased in YH-supplemented donkeys, highlighting improved mammary gland synthetic capacity and nutrient partitioning. Milk fat and protein are core determinants of milk nutritional value and directly influence energy and amino acid supply to nursing offspring [39,40,41]. The selective elevation in these components suggests that YH primarily targets metabolic pathways related to energy density and functional nutrient output rather than altering overall milk volume. Bioactive constituents of YH, including peptides, nucleotides, and mannan oligosaccharides, have been reported to enhance protein synthesis, lipid metabolism, and systemic energy utilization, which may collectively facilitate the synthesis and deposition of milk fat and protein in the mammary gland [38,42,43,44]. Although milk lactose and urea nitrogen levels were unaffected, the enrichment of milk fat and protein underscores the role of YH in optimizing mammary metabolic efficiency and improving the nutritional foundation available to foals during lactation.

The fecal microbiota analysis demonstrated that maternal dietary YH supplementation was associated with changes in the relative abundance of several bacterial taxa in donkey foals, whereas no significant differences were observed in overall microbial diversity. These findings suggest that maternal YH supplementation may selectively influence specific bacterial populations without substantially altering the overall microbial community structure. Similar observations have been reported in previous studies, in which dietary interventions modified the abundance of individual taxa while having limited effects on overall microbial diversity [45,46]. Notably, foals in the OYE group exhibited enrichment of genera such as Prevotella, Alistipes, Olsenella, and Proteiniclasticum. Previous studies have associated these genera with carbohydrate utilization, amino acid metabolism, and lipid metabolism in different animal species [47,48,49,50,51,52,53,54]. However, because the present study was based solely on 16S rRNA gene sequencing, no direct information regarding microbial metabolic activity or functional capacity was obtained. Therefore, the biological significance of these taxonomic changes should be interpreted with caution, and the observed differences should be regarded as associations rather than evidence of functional alterations. Likewise, although maternal YH supplementation was associated with changes in milk composition, selected serum biochemical parameters, and fecal bacterial composition, the present data do not establish causal relationships among these variables. Consequently, it cannot be concluded that the observed microbial changes mediated the serum biochemical responses or resulted directly from changes in milk composition. Future studies incorporating microbial metabolomics, short-chain fatty acid quantification, metagenomic or metatranscriptomic analyses, together with more comprehensive molecular characterization of milk composition, are warranted to clarify the functional significance of these microbial alterations and the mechanisms underlying the maternal dietary effects observed in donkey foals.

## 4. Materials and Methods

### 4.1. Animal Ethics Statement

The utilization of animals in this study adhered to rigorous ethical standards and was formally approved by the Animal Care and Use Committee of China Agricultural University (Approval No: AW90605202-1-05).

### 4.2. Experimental Design

A total of 16 healthy Dezhou jennies (2–3 years of age; body weight 256.52 ± 27.59) and their corresponding 16 Dezhou donkey foals with uniform body condition were enrolled in this study. All animals were managed under standardized husbandry conditions at Shandong Dong’e Ejiao Co., Ltd. (Liaocheng, Shandong, China). The jennies were housed individually in semi-open pens and fed a farm-formulated basal diet ad libitum twice daily at 07:00 and 19:00, with unrestricted access to clean drinking water throughout the experimental period. No probiotics or antibiotics were administered to any animals for at least three months prior to the trial and during the experiment. Following a 3-day adaptation period to stabilize feed intake, the jennies were weighed and randomly allocated to one of two dietary treatments (*n* = 8 per group). The experimental period lasted 60 days. The control group received the basal diet alone (Table S1), whereas the yeast hydrolysate group was fed the same basal diet supplemented with 0.5 g/kg yeast hydrolysate (DSM; Beijing DSM Biological Products Co., Ltd., Shanghai, China). Foals born to jennies in the control group were assigned to the OCON group, while foals born to jennies receiving yeast hydrolysate supplementation were assigned to the OYE group. Throughout the experimental period, growth performance parameters and diarrhea incidence were continuously monitored and recorded for both jennies and their foals.

### 4.3. Sample Collection

On the final day of the experimental period, all donkey foals were weighed in the morning following an overnight fast prior to feeding. Subsequently, approximately 5 mL of blood was collected from the jugular vein of each foal into sterile vacuum tubes before the morning meal. Blood samples were centrifuged at 3000× *g* for 10 min at 4 °C to separate serum. The obtained serum was immediately snap-frozen in liquid nitrogen and stored at −80 °C until further biochemical analysis. Fresh fecal samples were aseptically collected from the perianal region of each foal using sterile gloves to minimize environmental contamination. Samples were transferred into sterile 2 mL centrifuge tubes, immediately frozen in liquid nitrogen, and transported to the laboratory on dry ice. Upon arrival, all fecal samples were stored at −80 °C prior to microbial community analysis.

### 4.4. Serum Biochemical Indices

Serum biochemical parameters, including alanine aminotransferase (ALT), aspartate aminotransferase (AST), alkaline phosphatase (ALP), total protein (TP), albumin (ALB), total cholesterol (TC), triglycerides (TG), high-density lipoprotein cholesterol (HDL-C), and low-density lipoprotein cholesterol (LDL-C), were measured using an automated biochemical analyzer with commercially available assay kits (Nanjing Jiancheng Bioengineering Institute, Nanjing, China), strictly following the manufacturer’s instructions.

### 4.5. Milk Composition Analysis

Milk samples were collected from jennies in the control group and the yeast hydrolysate-supplemented group, with six biological replicates per treatment. Milk sampling was conducted during the morning milking period. Approximately 50 mL of milk was collected aseptically from each jenny into sterile containers after discarding the first few streams to avoid contamination. Milk samples were immediately cooled to 4 °C and transported to the laboratory for analysis. The concentrations of milk fat, milk protein, lactose, urea nitrogen, and somatic cell count (expressed as ×10^3^ cells/mL) were determined using a CombiFoss™ 7 milk analysis system (FOSS, Hillerød, Denmark) according to the manufacturer’s instructions. All measurements were performed in duplicate, and quality control procedures were applied using standard reference samples to ensure analytical accuracy and repeatability.

### 4.6. Microbiota Sequencing and Analysis

Total DNA was extracted from 200 mg of each fecal specimen using the E.Z.N.A.^®^ Soil DNA Kit (Omega Bio-tek, Norcross, GA, USA) according to the manufacturer’s protocol. The extracted DNA was used as a template to amplify the V3–V4 hypervariable region of the bacterial 16S rRNA gene using universal primers 338F (5′-ACTCCTACGGGAGGCAGCA-3′) and 806R (5′-GGACTACHVGGGTWTCTAAT-3′). The amplified products were verified by 2% agarose gel electrophoresis, purified using the AxyPrep DNA Gel Extraction Kit (Axygen Biosciences, Union City, CA, USA), quantified with a Qubit 2.0 Fluorometer (Thermo Fisher Scientific, Waltham, MA, USA), and pooled in equimolar concentrations. The amplicon libraries were then sequenced on an Illumina MiSeq PE300 platform (Illumina, San Diego, CA, USA) according to the standard protocol of Majorbio Bio-Pharm Technology Co., Ltd. (Shanghai, China).

### 4.7. Statistical Analysis

All data are presented as the mean ± standard deviation (SD). Comparisons between the two experimental groups were performed using a two-tailed unpaired Student’s *t*-test. Statistical analyses and data visualization were conducted using GraphPad Prism 9.5 (GraphPad Software, San Diego, CA, USA). Differences were considered statistically significant at *p* < 0.05.

Raw Illumina sequencing reads were quality-filtered, denoised, merged, and checked for chimeras using the QIIME2 pipeline to generate high-quality datasets, with at least 50,000 valid sequences retained per sample for subsequent analyses. Amplicon sequence variants (ASVs) were inferred using the DADA2 algorithm. Taxonomic assignment of ASVs was performed using the RDP classifier (version 2.2) against the SILVA reference database. Alpha diversity indices, including ACE, Chao1, Shannon, and Sobs, were calculated using QIIME2 together with the vegan package in R (version 2.5.6). Beta diversity was assessed by principal coordinate analysis (PCoA), and differences in microbial community structure between groups were evaluated using permutational multivariate analysis of variance (PERMANOVA, Adonis) implemented in R (version 3.2.1). Differentially abundant bacterial taxa were identified using linear discriminant analysis effect size (LEfSe), with an LDA score ≥ 2.0 and *p* < 0.05 considered statistically significant. Venn diagrams illustrating shared and unique ASVs between groups were generated using the VennDiagram package in R (version 3.1.1). Functional prediction of genus-level microbial profiles was performed based on the KEGG Orthology database.

## 5. Conclusions

In conclusion, maternal dietary supplementation with yeast hydrolysate increased milk fat and milk protein contents and was associated with changes in selected serum biochemical parameters and the relative abundance of specific fecal bacterial taxa in donkey foals. Although foals from the YH-supplemented group exhibited a tendency toward increased back skin thickness, no significant differences were observed in body weight, neck skin thickness, or overall fecal microbial diversity. These findings provide preliminary evidence that maternal dietary yeast hydrolysate supplementation may influence selected physiological and microbial characteristics in donkey foals. However, given the relatively small sample size and the exploratory nature of the microbiota analysis, the present study cannot establish causal relationships or elucidate the mechanisms underlying these associations. Future studies involving larger cohorts, comprehensive molecular characterization of milk composition, and functional analyses of the gut microbiota are warranted to validate these findings and further clarify their biological significance.

## Data Availability

The raw sequencing data generated in this study have been deposited in the NCBI Sequence Read Archive (SRA) under accession number PRJNA1477230. All other data supporting the findings of this study are included in the article and its Supplementary Materials. Additional information is available from the corresponding author upon reasonable request.

## Supplementary Materials

The following supporting information can be downloaded at: https://www.mdpi.com/article/10.3390/ijms27167171/s1.

## Abbreviations

The following abbreviations are used in this manuscript:

ABC	ATP-binding cassette
ACE	Abundance-based coverage estimator
ALB	Albumin
ALP	Alkaline phosphatase
ALT	Alanine aminotransferase
AST	Aspartate aminotransferase
ASVs	Amplicon sequence variants
DADA2	Divisive Amplicon Denoising Algorithm 2
DNA	Deoxyribonucleic acid
HDL-C	High-density lipoprotein cholesterol
KEGG	Kyoto Encyclopedia of Genes and Genomes
LC–MS/MS	Liquid chromatography–tandem mass spectrometry
LDA	Linear discriminant analysis
LDL-C	Low-density lipoprotein cholesterol
LEfSe	Linear discriminant analysis effect size
MCON	Control group fed the basal diet
MYE	Yeast hydrolysate supplementation group
OPLS-DA	Orthogonal partial least squares discriminant analysis
PCoA	Principal coordinate analysis
PCR	Polymerase chain reaction
PERMANOVA	Permutational multivariate analysis of variance
QIIME	Quantitative Insights Into Microbial Ecology
RDP	Ribosomal Database Project
SD	Standard deviation
Sobs	Observed species
SPSS	Statistical Package for the Social Sciences
TC	Total cholesterol
TG	Triglyceride
TP	Total protein
UCG	Uncultured group
UHPLC	Ultra-high-performance liquid chromatography
VIP	Variable importance in projection
YH	Yeast hydrolysate
16S rRNA	16S ribosomal RNA
