# Supplementary material for: Maternal Dietary Yeast Hydrolysate Alters Milk Composition and Influences Metabolic Status and Gut Microbiota in Dezhou Donkey Foals
Source: Int J Mol Sci. 2026 Aug 11;27(16):7171. doi: 10.3390/ijms27167171 (PMC13513465; doi:10.3390/ijms27167171)
Supplement: Supplementary file 1 [file ijms-27-07171-s001.zip › ijms-4429856-supplementary.pdf]

Table S1. Composition and nutritional level of experimental concentrate (dry-matter basis).

Ingredients	Content (%)	Nutrients	Nutrient level (%)
Corn	31.75	Dry matter	88.53
Middlings bran	12.00	Crude protein	17.18
Wheat flour middling	21.00	Ash	8.37
DDGS	5.00	Crude fber	4.27
Wheat bran	18.00	Ether extract	4.59
Soybean meal	6.65	Calcium	1.27
NaCl	0.63	Phosphorus	0.52
$CaCO_3$	3.72	Lysine	0.75
$CaHPO_4$	0.25		
Premix1	1.00		
**Total**	100.00		

[1] Premix/kg: VA, 600 KIU; VD_3, 125 KIU; VE, 3500 IU; Fe, 2 g; Cu, 800 mg; Zn, 6 g; Mn, 13 g; I, 95 mg; Se, 50 mg.
